# Assessment of nitrate-N contamination in the Chunnakam aquifer system, Jaffna Peninsula, Sri Lanka

**DOI:** 10.1186/2193-1801-3-271

**Published:** 2014-05-30

**Authors:** Meththika Vithanage, Thushyanthi Mikunthan, Selverajah Pathmarajah, Sutharsiny Arasalingam, Herath Manthrithilake

**Affiliations:** Chemical and Environmental Systems Modeling Research Group, Institute of Fundamental Studies, Kandy, Sri Lanka; Department of Agriculture Engineering, University of Jaffna, Jaffna, Sri Lanka; Department of Agriculture Engineering, University of Peradeniya, Peradeniya, Sri Lanka; International Water Management Institute, Sunil Mawatha, Battaramulla, Sri Lanka

**Keywords:** Nitrate, DRASTIC, Agricultural pollution, Nitrogen fertilizer

## Abstract

Jaffna peninsula in Sri Lanka is an area of intensive agriculture using extensive organic and inorganic nitrogenous compounds and hence, this study was focused on assessing vulnerability of karstic aquifer system with specific focus on nitrate contamination, and compare loads of nitrate from agriculture. The total number of the wells sampled in the Chunnakam aquifer is 44. The coverage of wells with measurements of nitrate and nitrite concentrations in the database covering the study period from Januray, 2011 to August, 2011. The intrinsic vulnerability of the area is estimated by the DRASTIC model and the modified DRASTIC method was used to determine the nitrate-specific vulnerability of the aquifers. Average concentrations of nitrate-N and nitrite-N during the study period were 4.869 and 0.014 mg/L respectively. The average number of wells exceeding permissible level of NO_3_–N is approximately 6–12, which means that about 14-28% out of the 44 wells. Modified DRASTIC (DI) index value computed as explained above increased from DI = 177 to a range of 182 to 197. In spite of the increase, the Modified DI values show that the aquifer vulnerability specific to nitrate contamination remains in “high” category. Although nitrogen loading at the domestic sources and irrigation is of the same order of magnitude, the loading from fertilizer input is much larger which is about 15 times higher. This finding suggests that the fertilizer input in agricultural areas constitute a significant contribution to the nitrogen content in the groundwater and soils in agricultural areas of Jaffna.

## Introduction

Although Nitrogen input is essential for high crop yields, an excess use of N fertilizer cannot promise a substantial increase in crop productivity. Overuse of nitrogen fertilizer results in diminishing crop returns (Tilman et al. 2002) and leads to diminished environmental quality and human wellbeing (Galloway et al. 2003; Liu and Diamond 2005). Strong correlations between the recharge and land use have been observed indicating the control of patterns of land use on groundwater quality, especially in terms of nitrate (Jayasekara et al. 2011). More recently, the contribution of groundwater nitrogen to surface-water nutrient budgets also has been recognized (Valiela et al. 1990). Similarly, studies have shown the potential of vulnerability assessment modeling for supporting decision making processes to protect and manage groundwater aquifers (Mastrocicco et al. 2011; Jayasekara et al. 2011). China, now the largest consumer of synthetic N in the world, accounts for 32% of the world’s total consumption (Heffer 2009).

Overuse of synthetic nitrogen fertilizers has become widespread across Sri Lanka, similar to that of some other countries, resulting severe environmental problems (Jeyaruba and Thushyanthi 2009; Liyanage et al. 2000; Jayasekara et al. 2011). Nitrate-N content in drinking water supply wells found in very high concentrations and ranged from 7.1 to 15.3 mg/L in Jaffna (Jeyaruba and Thushyanthi 2009), and in 56% of 225 groundwater samples taken in the Kalpitiya area (Liyanage et al. 2000). If the excessive application of nitrogen fertilizer is not brought under control, Sri Lanka’s waters will continue to deteriorate. Intensive rehabilitation and development activities are ongoing in Jaffna after the end of the 30 years long civil war. People return to their lands and extensive agricultural activities are enduring throughout the peninsula. Farmers in Jaffna are tend to overuse agrochemicals due to the loss by the limestone with large cavities in the subsurface. High population density may also contribute to N contamination of groundwater in Jaffna Peninsula by the pit latrines in the limestone strata. Hence, the groundwater contamination due to high nitrates will continue to rise. Few studies conducted on nitrate contamination of groundwater in Jaffna aquifer system have shown high concentrations (Jeyaruba and Thushyanthi 2009) which may be a cause of the high incidence of cancer in Jaffna Peninsula (Sivarajah 2003). A five year study conducted on the geographical pathology of malignant tumor in Sri Lanka showed the highest incidence (184 per 100,000 populations) in the biopsy material among the nine provinces of Sri Lanka is the Northern Province (Panabokke 1984). With the underlying limestone, Jaffna aquifer system considered to be vulnerable to pollution (Panabokke and Perera 2005). Hence, it may be important to distinguish the different sources of nitrate input and budgeting will give an understanding about the sources to be managed, which have not been focused by earlier studies.

In contrast to developed countries, developing countries as Sri Lanka often lack of proper policy and institutional framework to manage their goundwaters, which may affect severely in the long run (Villholth and Rajasooriyar 2009). Without clear monitoring and assessment, this situation may become worse. However, no proper assessment of nitrogen budgeting has been conducted for Jaffna peninsula although studies have supported that the groundwater nitrate concentrations in Jaffna peninsula are beyond the permissible levels. Considering all above facts, the purpose of this study was to investigate the threats to groundwater in the Jaffna aquifers, where intensive use of agrochemicals are in use, with specific focus on nitrate budgeting in agricultural lands and domestic areas, assess vulnerability of the aquifers to nitrate contamination using simple model calculations, and compare loads of nitrate from agriculture with domestic input. Specifically we assessed the spatial and temporal variation in nitrate in agricultural and domestic wells and mapped using GIS. The intrinsic vulnerability of the area was estimated using the modified DRASTIC method and determined the nitrate-specific vulnerability of the aquifers. It is anticipated that the findings from this study will provide insight into the complex water quality issues of the limestone aquifers and therefore, the results and conclusions drawn can be used in implementing effective governance and public policy.

## Methodology

### Study area

**Figure 1:**
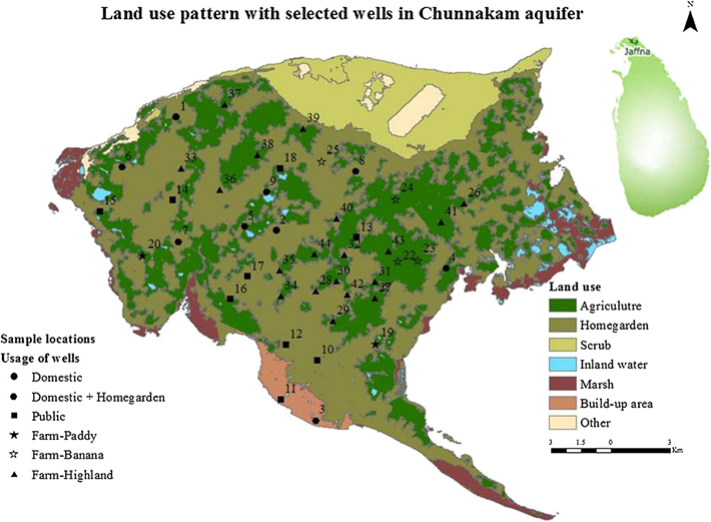
**The physical layout of Jaffna peninsula, its land use and monitoring well locations.**

The Jaffna Peninsula is situated in the Northern extreme of Sri Lanka. It is geographically confined to the North and East by the Indian Ocean and on the West by the Palk Strait, and the Southern areas extend into the mainland of the country. The Jaffna District occupies an extent of 1,023 square kilometers (km^2^) which includes inland waters. The Jaffna Peninsula, which is part of the dry zone in Sri Lanka, is underlain mainly by a Miocene limestone that is considered to be a good aquifer for groundwater storage and discharge. Most of the peninsula is used as home gardens and for agricultural activities (Figure 1). However, the region experiences groundwater problems, as the resource is limited and its quality has deteriorated over the years (Mikunthan and De Siva 2010). Groundwater is the only source of water for the entire Peninsula and there are currently no major water supply schemes. High evapotranspiration loss during the dry season and high run-off loss during the wet season play a major role in determining the limited storage of groundwater in the Peninsula.

Rainfall acts as a major source of groundwater recharge, and its seasonality and variability greatly affects the quantity and quality of groundwater. The major rainy season occurs during October to December due to the northeast monsoon, and the minor rainy season occurs during April to May due to the southwest monsoon. The period between the southwest and northeast monsoons is dry and this dryness extends from June to September. The months of September/October to January/February and February/March to August/September are referred to as *Maha* (wet season) and *Yala* (dry season), respectively. The bulk of the rainfall is received during the months from October to January, with little or no rainfall afterwards. Of the total annual average rainfall, 80% of the rainfall occurs during the northeast monsoon.

### Analysis

The nitrate concentration data used in this study were obtained entirely from the laboratory analysis and questionnaire survey. All available data were assembled into a single composite database to facilitate the analysis. The total number of the wells sampled in the Chunnakam aquifer was 44 (Figure 1). The coverage of wells with measurements of nitrate and nitrite concentrations in the database was conducted from Januray, 2011 to August, 2011.

### Groundwater vulnerability assessment based on Nitrogen

#### Nitrogen budgeting

For nitrogen budget calculations, characterization of nitrogen sources and identification of areas with heavy nitrogen loadings from point and non-point sources is essential. The conceptual model of nitrate fate and transport in groundwater integrates several components: (i) spatial distribution of on-ground nitrogen loadings; (ii) detailed assessment of all nitrogen sources in the study area (iii) and nitrate in groundwater (Almasri and Kaluarachchi 2004; Ledoux et al. 2007; Almasri 2007). Accurate nitrate budgeting was difficult due to the complex interactions between land use practices, on-ground nitrogen loading, groundwater recharge, soil nitrogen dynamics, and soil characteristics. The modeling framework accounts for point and non-point sources of nitrogen. This integration was of great importance to realistically account for the different processes that nitrogen undergoes and in order to arrive at rational estimates of nitrate concentrations in groundwater.

#### Use of DRASTIC

DRASTIC is an acronym for an empirical model with seven variables namely: Depth to the groundwater table, net groundwater recharge, Aquifer media, Soil media, Topography, Impact of vadose zone media, and hydraulic conductivity of the aquifer (Aller et al. 1985). The model DRASTIC, was developed by Aller et al. (1985). In this study, DRASTIC was used together with modified DRASTIC to assess groundwater vulnerability of Chunnakam aquifer system in Jaffna. This method is considered as a standardized method for evaluating groundwater vulnerability to contamination and has been used in the world (Fritch et al. 2000; Shukla et al. 2000; Al-Zabet 2002). The DRASTIC method has also been applied in many different climates including Sri Lanka (Babiker et al. 2005; Werz and Hötzl 2007; Jayasekara et al. 2011).

A vulnerability assessment provides the intrinsic vulnerability of a given region to potential contamination using hydrologic and recharge properties independent of a contaminant (Jayasekara et al. 2011). In DRASTIC, each of the hydrogeologic factors was assigned a rating from 1 to 10 based on a pre-set range of values. The weight assigned by Aller et al. (1985) to each variable is as follows: depth to water table and impact of vadose zone, 5; net recharge, 4; aquifer media and hydraulic conductivity, 3; soil media, 2; and topography, 1. The DRASTIC Index (DI) is given as *DI* = *DwDr* + *RwRr* + *AwAr* + *SwSr* + *TwTr* + *Iw Ir* + *CwCr* (1) where *Dw*, *Rw*, *Aw*, *Sw*, *Tw*, *Iw*, and *Cw* were the weights allocated to depth, recharge, aquifer media, topography, impact, and conductivity, respectively. Similarly, *Dr*, *Rr*, *Ar*, *Sr*, *Tr*, *Ir*, and *Cr* were the ratings allocated to depth, recharge, aquifer media, topography, impact, and conductivity, respectively.

The intrinsic vulnerability of the area was estimated by the DRASTIC. Aller et al. (1985) defined DRASTIC qualitative index categories for vulnerability as: 1–100, low; 101–140, moderate; 141–200, high; and *>*200, very high. The depth to the groundwater table was measured at the same 44 well locations where sampling was performed. The net recharge was estimated based on the previous observations.

#### Modified DRASTIC method

The modified DRASTIC method (Liang et al. 2009; Nobre et al. 2007) was also used in this study to determine the nitrate-specific vulnerability of the aquifers. Assigned ratings and weights to the on ground nitrogen loading are then added to the final DRASTIC index values obtained using Eq. 1 to produce a composite index of groundwater vulnerability by nitrate.1

where CDI is the composite DRASTIC index, and *Nw* and *Nr* are the weight and rating given to the total on-ground nitrogen loading. The total on-ground nitrogen loading consists of two parts; agricultural loading due to fertilizer and non-agricultural loading due to sanitation and human waste. The human and sanitation nitrogen loading of 14.3 g per capita per day was estimated assuming the ammonia concentration is negligible based on Jayasekara et al. (2011). Agricultural nitrogen loadings were estimated using the fertilizer and irrigation nitrogen loadings observed through on-site measurements and interviews. The ratio of nitrogen content by weight in each fertilizer type and the total amount of fertilizer applied by each fertilizer type for different crops in different land uses was estimated through the results from the questionnaire survey.

## Results and Discussion

### Nitrogen distribution across the Chunnakam groundwater

**Figure 2:**
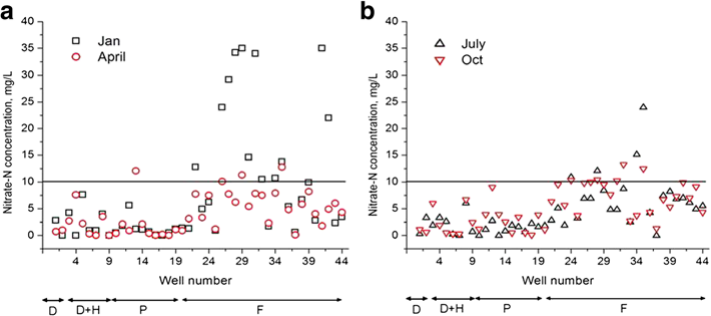
**Spatial and temporal variation of Nitrate-N in Jaffna aquifer system based on well use; a) January and April; b) July and October.** D; Domestic wells, D + H; Domestic wells serving home gardens, P; Public wells and F is for farm or agricultural wells (agro wells).

**Figure 3:**
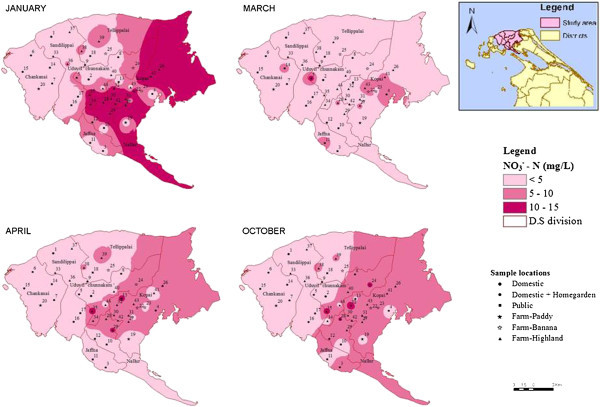
**Geochemical maps of Nitrate-N in the study area.**

Average concentrations of nitrate-N and nitrite-N for the whole year and for the entire area during the study period were 4.869 and 0.014 mg/L respectively. The average number of wells exceeding permissible level of NO_3_–N, the Sri Lanka Standard Institute (SLSI) drinking water guideline of 10 mg/L, is approximately 6–12 depending on the time of the year, which means that about 14-28% out of the 44 wells. The nitrate-N concentration ranged from 0 to 35 mg/L. All values from domestic, domestic with home garden and public wells were acceptable for drinking purposes during the end of the wet season as the nitrate-N values were below the limit of permissible level (10 mg/L). Among the farm wells monitored, 38% exceeded the limit of 10 mg/L and were not suited for drinking purposes.The spatial and temporal fluctuations of nitrate-N in wells under different usages are shown in Figures 2 and 3 for the study period. The ranges of nitrate-N observed in domestic, domestic with home garden and public wells were around 0–12.1 mg/L throughout the year. In all of the other domestic and public wells, the temporal variation of nitrate N was below 10 mg/L throughout the year. Normally in home gardens, inorganic fertilizers were not used and the abstraction levels and the amount of irrigation are also less than farm wells.Groundwater within the intensively cultivated area had high levels of nitrate-N concentrations. High nitrate-N concentration in groundwater was observed during January (Figure 2). Concentration of nitrate-N in paddy and banana land use had lower values than highland crops (crops such as carrot, cabbage etc. grown in elevated altitudes). Nitrate-N found in most of the wells in highland crop land use exceeded the recommended Sri Lankan standard level for drinking purposes. Even though these wells are used for agricultural purposes, people who are working in the field use the well water for drinking. A general decreasing trend in nitrate-N concentrations were observed from January to March.

### Nitrogen Loading at the Surface

As mentioned above, a comprehensive survey of the fertilizer amounts used in various farms according to the crop type was used to determine the surface loading of nutrients. A variety of inorganic fertilizer types are used in the study area. The amount of surface loading depends on the nitrogen content of the fertilizer. Based on the fertilizer type (both inorganic and organic), an estimate of the nitrogen content was made. Using this information, the total surface loading of nitrogen for areas served by agricultural wells was computed. A summary of results from the N-loading computation is shown in Table 1.

**Table 1:** **Total surface loading of N in areas served by agricultural wells**

Agro well	Crop type	Area (sq.m)	Total N-load (kg)
C2			
	Onion	1764	35
	Pumpkin	1764	25
	Tobacco	1764	175
D1			
	Onion	3528	164
D2			
	Beet root	441	330
	Onion	441	10
	Pumpkin	882	14
	Tobacco	1323	43.75
	Tomato	441	13.75
D5			
	Amarathus	504	1
	Beans	504	1
	Cassava	2016	175
	Snake gourd	504	0
D6			
	Beet root	3326.4	358.25
	Chilli	2520	56.25
	Onion	1260	33.125
E2			
	Beet root	3528	130
	Onion	441	13.75
E5			
	Beet root	2419.2	226.5
	Onion	604.8	7.2
	Tobacco	1209.6	150
E6			
	Banana	2016	100
F5			
	Banana	1008	0
	Leeks	403.2	7.5
	Onion	604.8	106.3
	Tobacco	1008	15
G4			
	Beet root	4032	86
	Onion	504	0
	Tobacco	2016	140
G5			
	Onion	6753.6	493.1
NW1			
	Banana	1512	75
NW10			
	Tobacco	2016	268
NW2			
	Banana	2520	
	Onion	2016	240
NW3			
	Banana	2520	250
	Beet root	1008	15
NW4			
	Banana	1209.6	0
	Cabbage	1209.6	63
	Pumpkin	1209.6	8.4
NW5			
	Amarathus	705.6	3.5
	Onion	604.8	7.5
	Tobacco	1512	217.5
NW8			
	Cassava	2016	50
NW9			
	Capsicum	504	0
	Chilli	504	0
	Tobacco	2520	193.75
	Total	73117.8	4302.125

Using the area served by the agricultural wells (Agro wells), nitrogen loading per square kilometers was calculated and compared with the second major source nitrogen loading which was domestic waste. The surface loading of nitrogen over the study area was equivalent to about 58.8 metric tons/km^2^ for the period of study. Assuming the ammonia content to be negligible, Jayasekara et al. (2011) reported a unit value of 14.3 g/per capita per day for the total nitrogen content in human and kitchen waste. Using this figure and a population density of 790 persons/km^2^, the surface loading of nitrogen from domestic sources was computed yielding an average figure of 4.1 metric tons/km^2^ per day. Finally, the other known source of nitrogen loading at the surface was irrigation. The average abstraction from farm wells for agricultural activities varies from 13 m^3^/d to 19 m^3^/d (Jeyaruba and Thushyanthi 2009). For the 19 agricultural sites corresponding to the sampling wells it was assumed that they were irrigated for 100 days a year. The average concentration of NO_3_-N in the 19 wells was computed by using the water quality data as 8.32 mg/L. Using these assumptions, the surface loading from irrigation with the study site was computed as 506 kg/year which is equivalent to 3.46 metric tons/km^2^.

The surface loading figures corresponding to the fertilizer input, domestic sources (sewage and kitchen waste etc.) and irrigation showed an important contrast. The nitrogen loading at the surface for the domestic sources and irrigation was of the same order of magnitude. However, the loading from fertilizer input was much larger which was about 15 times higher than the other two sources. This finding suggested that the fertilizer input in agricultural areas constitute a significant contribution to the nitrogen content in the groundwater and soils in agricultural areas of Jaffna.

**Figure 4:**
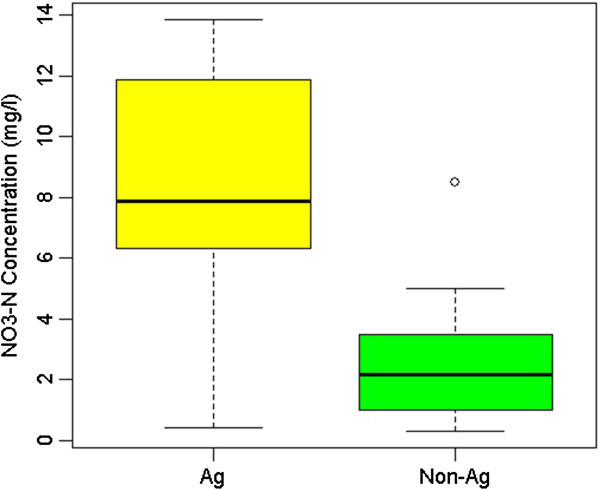
**Box and Whisker plots of NO**_**3**_ **-N for agricultural and non-agricultural wells.**

The effect of high nitrogen input through fertilizer application was observed in the NO_3_-N data obtained from sampling the wells in the study area. Figure 4 shows the box-whisker plots of NO_3_-N for both agricultural wells and non-agricultural wells. It was clear that the NO_3_ levels for the agricultural wells were about four times higher than the same levels for non-agricultural wells. The heavy fertilizer usage for agriculture in Jaffna region appears to have resulted in elevated levels of NO_3_ levels in groundwater in the region and the resulted concentrations at times were higher than guidelines established by WHO which is 10 mg/L for NO_3_-N.

### Groundwater Vulnerability

Depth to water table was determined from the field observations. Net recharge was computed using the rainfall observations for the 2007–2011 period and an estimate of the fraction of rainfall-recharge. The average rainfall for this period was 1539 mm. Groundwater recharge rates of the limestone aquifer range from 23% to 25% of annual rainfall as determined by the Modified Soil Moisture Balance (Rushton et al. 2006) for the same period. This was confirmed by another model, Water Table Fluctuation (WTF) method which provides estimates of 26% to 29%. Groundwater recharge using the soil moisture balance model for ten years from 1971 to 1980 in five agrarian service centre was estimated by (Jeyaruba and Thushyanthi 2009; Thiruchelvam et al. 1994) and the results showed that 31 to 33% of the rainfall was recharged into the aquifer. Further he reported that on average about 33% of the total rainfall is recharged into the aquifer. For DRASTIC calculations, an average recharge rate of 30% was assumed. Jaffna peninsula consists largely of karstic limestone and therefore the aquifer media in DRASTIC was assumed to be limestone. The category of soil media available in DRASTIC tables closest to the study region was silt loam. The general topography of the study region has a slope of about 2% and that was used as the Topography parameter in DRASTIC. The impact of vadose zone media was determined from DRASTIC tables by assuming that the vadose zone consists of sand and gravel with significant silt and clay. Finally since there were no measurements of hydraulic conductivity, it was assumed that it is very high for the karstic system in Jaffna. The hydraulic conductivity values were assumed to be over 2000 gpd/ft^2^ which is at the upper end of the scale in DRASTIC for that variable.

The intrinsic vulnerability index that was calculated based on the assumptions and data provides a basic understanding about the status of the aquifer in terms of susceptibility. The DRASTIC index corresponding to estimated values is 177. This was a composite value for the entire study region. Based on the qualitative index categories for vulnerability, the computed DRASTIC index was in the “high” category. It follows that the study region has a high intrinsic vulnerability for contamination. This high level of vulnerability is resulted from various factors as seen from the ratings given in Table 2. A combination of factors involving shallow water table, high net recharge, limestone aquifer, low topography, and the high hydraulic conductivity make Jaffna peninsula highly vulnerable for contamination.

**Table 2:** **Estimates of the seven variables used for computing the DRASTIC index**

DRASTIC index	Variable	Estimate	Rating	Weight
D	Depth to water table	5-15 feet*	9	5
R	Net recharge	18.2 inches^*^	9	4
A	Aquifer media	Limestone	6	3
S	Soil media	Silt Loam	4	2
T	Topography	2%	10	1
I	Impact of vadose zone media	Sand and gravel with significant silt and clay	6	5
C	Hydraulic Conductivity	>2000 gpd/ft^2*^	10	3

*As DRASTIC requires variables in English Units, estimates in SI Units were converted.

Specific vulnerability of the aquifer attributable to nitrate was determined by using the Modified DRASTIC index. Basically, the modified index was calculated by adding a nitrate specific quantity, N_r_N_w_ to the regular DRASTIC index where N_r_ is the rating corresponding to nitrogen loading and N_w_ was the associated weight. The surface loading of nitrate-nitrogen is available from Table 1 and following Jayasekara et al. (2011) the ratings with a range of 1 to 5 were calculated by using equal loading intervals. In addition, the weight for nitrogen loading was assumed to be 5 for the calculation of Modified DRASTIC index which ranged from 182 to 197. This value is somewhat higher than the DRASTIC index reported above indicating the added pollution potential from nitrate.

## Conclusions

Modified DRASTIC (DI) index value computed as explained above increased from DI = 177 to a range of 182 to 197. In spite of the increase, the Modified DI values showed that the aquifer vulnerability specific to nitrate contamination remains in “high” category. However, the high end of Modified DRASTIC index was closer to the threshold value for transiting the vulnerability from the “high” category to a “very high” category. Many of the wells in agricultural fields contained high concentrations of nitrate which exceeded the permissible levels for drinking water. Although the nitrogen loading estimations at the surface for the domestic sources and irrigation were similar in magnitude, the loading from fertilizer input was about 15 times higher than the domestic and irrigation sources. In conclusion, the aquifer system in Jaffna remains highly vulnerable to nitrate specific contamination. The risk of contamination is largely attributed to the heavy fertilizer use for agriculture in the area.
